# Platelet inhibition delays time to neurosurgical intervention for cerebral metastasis in non-small cell lung cancer

**DOI:** 10.1038/s41598-025-25708-8

**Published:** 2025-11-04

**Authors:** David Wasilewski, Julia Onken, Sae-Yeon Won, Artem Rafaelian, Adrian Hempelmann, Joshua Bernstock, Claudia Maletzki, Thomas Freiman, Peter Vajkoczy, Florian Gessler, Daniel Dubinski

**Affiliations:** 1 Department of Neurosurgery, Charité-Universitätsmedizin Berlin, Berlin, Germany; 2 Department of Neurosurgery, Rostock University Medical Center, Rostock, Germany; 3 Department of Internal Medicine, Medical Clinic III-Hematology, Oncology, Palliative Medicine, Rostock University Medical Center, 18057 Rostock, Germany; 4 Department of Neurosurgery, Brigham and Women’s Hospital, Harvard Medical School, Boston, MA USA

**Keywords:** Aspirin, Platelet function, Cerebral metastases, Survival analysis, Cerebral metastasis prevention, Non-small-cell lung cancer, Metastasis

## Abstract

An inverse association between the use of platelet inhibitors and the risk of cancer has been reported by numerous epidemiological studies in the past. The effects of antiplatelet agents on the cerebral metastasis formation of non-small cell lung cancer (NSCLC) are largely unknown. We therefore, investigated the effect of platelet inhibition in NSCLC patients at the time of the first diagnosis of cerebral metastases. We retrospectively investigated the clinical course of 417 NSCLC patients with cerebral metastases who underwent craniotomy for metastasis resection during the course of their disease. The presence of platelet inhibition prior to cerebral metastases diagnosis was used to dichotomize the cohort. Relevant clinical parameters, time to neurosurgical intervention for cerebral metastases, overall survival, and the incidence of intracranial hemorrhage or hemorrhagic transformation of metastases, were compared between the two groups. The presence of platelet inhibitor intake was associated with a significantly prolonged time to neurosurgical intervention for cerebral metastases in non-small cell lung cancer 63 vs. 47 months; (*p* = 0.001). Furthermore, platelet inhibitor intake was also associated with an increased overall survival of 12 vs. 10 months (*p* = 0.02). Statistically, no increased risk of hemorrhagic transformation of the metastasis or intracranial hemorrhage was found (*p* = 0.635 and *p* = 1.000), respectively. In this retrospective study, the use of platelet inhibitors was not associated with an increased risk of intracranial hemorrhage, the use of platelet inhibitors was associated with delayed need for neurosurgical treatment for cerebral metastases and improved overall survival in NSCLC patients.

## Introduction

Non-small cell lung cancer (NSCLC) accounts for the majority of all lung cancer cases, and approximately 65% of patients present with locally advanced or metastatic disease at the time of diagnosis^1,2^. Among those, approximately 20% will have cerebral metastases at presentation and around 30% will develop cerebral metastases during the course of their disease. The pathomechanism of how NSCLC cerebral metastasis occurs and what influences the timing is currently undetermined^3^.

NSCLC cells were shown to induce platelet activation and aggregation leading to a coating process that protects circulating tumor cells from harmful effects of shear forces, shields tumor cells from the immune system, and provides growth factors^4,5^. More specifically, platelets have been found to promote tumor cell growth and metastasis by secreting multiple growth factors and inducing an epithelial-mesenchymal-like transition (EMT) and evade the immune surveillance by impairing NK cell cytotoxicity and inhibiting T cell immunity^6^.

Platelet inhibition on the other hand has gained momentum because of its potential anti-cancer effects supported by both in vitro and in vivo studies. Observational epidemiologic studies showed that aspirin, a nonselective cyclooxygenase (COX) inhibitor, commonly used in patients with cardiovascular disease prolongs survival in various cancers including NSCLC^7^.

The primary objective of this study is to investigate whether platelet inhibition can delay the onset of cerebral metastasis in NSCLC patients.

## Methods

### Patient selection

All patients who underwent surgical resection for brain metastasis at the neurosurgical department at the University Hospital Rostock and Charité-Universitätsmedizin Berlin between 2016 and 2022 were eligible to be included in the analysis. Indication for craniotomy were neurological deficit, metastasis size unsuitable for radiotherapy and tumor size > 3 cm in diameter. The indication for surgical treatment was confirmed in advance at an interdisciplinary tumour conference for all cases. 417 patients were included in the study. Patient characteristics and medical data were collected via the institution’s electronic database. For this retrospective analysis, ethical approval was obtained from the Ethics Committee of the University Medicine Rostock, Germany (Identification number: A 2021 − 0112); as a non-interventional bicentric, retrospective study, patient consent was waived.

### Clinical and radiological data

Patient medical charts were analysed by two neurosurgeons (D.W. and A.R.). Metastasis and edema volume were analysed by semi-automatic segmentation with IPlannet 3 (Cranial planning software, Brainlab AG, Feldkirchen, Germany). All metastasis segmentation was done semi-automatically with the ‘Smartbrush’ tool of the Brainlab Elements software. A two-dimensional segmentation was drawn in the axial image and a second two-dimensional segmentation was drawn in a coronal slide. These two segmentations automatically generated a three-dimensional graphic of the tumor. The three-dimensional graphic was then manually corrected by adding or erasing certain areas. Platelet inhibitors included Acetylsalicylic acid (Aspirin→), Clopidogrel (PlavixⓇ), Prasugrel (EffientⓇ), Ticagrelor (BrilintaⓇ) und Cangrelor (KengrealⓇ). Intracranial hemorrhage was defined as ≥ 10 ml in volume, required surgical intervention, or was associated with clinical symptoms, such as nausea and vomiting, or focal neurologic deficit.

To estimate the survival rates and cerebral metastasis rates, the Kaplan-Meier analysis was used. The differences between curves were assessed using the log-rank test. Time to neurosurgical intervention for cerebral metastases was defined as the time from NSCLC diagnosis to first neurosurgical intervention for metastasis resection. Overall survival (OS) was defined as the time of first presentation to death. Additionally, postoperative overall survival (OS-PO) was defined as the time from neurosurgical intervention to death.

### Statistics

Kaplan–Meier survival curve analysis and the log-rank test were conducted to obtain the median OS of the groups. Univariate and multivariate analyses were performed using a Cox proportional regression model. Hazard ratios (HRs) and 95% confidence intervals (CIs) were calculated. Visualization was performed with Biorender. Multivariate analysis was performed on variates with *p* values < 0.2, and *p* values < 0.05 were considered to indicate statistical significance. All statistical analyses were conducted using the GraphPad Prism 10 (GraphPad Software, California, USA). For patient characteristics, descriptive statistics were used.

## Results

### Cohort characteristics

The study group consisted of 417 patients, the average age was 64.4 years old (IQR 53–81) and 176 (42%) of the patients were female. Medical record of platelet inhibition showed a total of 74 patients (17.7%) cases. Of these, 72 patients were on monotherapy with acetylsalicylic acid (97.3%), one patient was on monotherapy with clopidogrel (1.35%), and one patient was receiving dual antiplatelet therapy (1.35%).

Singular cerebral metastasis was seen in 357 cases (85%) and multiple cerebral metastasis in 60 (15%) accordingly. The median volume of cerebral metastasis was 12 ml (IQR 8–15)) and 67.1 (IQR 57–73) for median volume of perilesional edema. Hydrocephalus was seen in 46 patients (11%). Hemorrhagic metastasis transformation was recorded for 87 patients (21%) and intracranial hemorrhage for 7 patients (2%). The median Karnofsky performance status (KPS) was 80 (IQR 70–90) and median Graded Prognostic Assessment (GPA) 2 (IQR 1–3). Table 1.

**Table 1:** Clinical features of NSCLC patients at the time of diagnosis of cerebral metastases. GPD: graded prognostic Assessment, KPS: Karnofsky performance status scale.

Patient characteristics	(*n* = 417)
Female, n (%)	176 (42)
Age, median (IQR)	64.9 (53–81)
Platelet inhibitor yes, n (%)	74 (18)
Singular cerebral metastasis, n (%)	357 (85)
Multiple cerebral metastasis, n (%)	60 (15)
Volume of cerebral metastasis in ml, median (IQR)	12 (8–15)
Volume of perilesional edema in ml, median (IQR)	67.1 (57–73)
Hydrocephalus, n (%)	46 (11)
Hemorrhagic transformation, n (%)	87 (21)
Intracranial hemorrhage, n (%)	7 (2)
KPS, median (IQR)	80 (70–90)
GPA, median (IQR)	2 (1–3)
Time to neurosurgical intervention for cerebral metastases in months, median	55
Overall survival postoperative in months, median	11.5

**Table 2:** Univariate analysis of juxtaposed characteristics according to platelet inhibitor intake in NSCLC patients at the time of cerebral metastases diagnosis.

Patient characteristics (*n* = 417)	platelet inhibitor (*n* = 74)	no platelet inhibitor (*n* = 343)	Univariable		
			OR	95% CI	*p*-Value
Female, n (%)	26 (35)	150 (36)	0.6	0.41–1.17	0.195
Age, median (IQR)	65.4 (10.9)	64.5 (10.7)		1.80–3.60	0.513
Singular cerebral metastasis, n (%)	63 (85)	294 (86)	0.9	0.47–1.93	1
Multiple cerebral metastasis, n (%)	11 (15)	49 (14)	1.0	0.51–2.12	1
Volume of cerebral metastasis in ml, median(IQR)	12 (21.2)	12.1 (19.9)		4.97–5.17	0.969
Volume of perilesional edema in ml, median (IQR)	65.7 (60.2)	67.7 (61.3)		14.74–18.74	0.814
Hydrocephalus, n (%)	13 (17)	33 (10)	2.0	0.99–4.02	0.061
Hemorrhagic transformation, n (%)	20 (27)	84 (24.5)	1.1	0.64–2.01	0.635
Intracranial hemorrhage, n (%)	1 (1)	6 (2)	0.7	0.09–6.48	1
Median KPS (SD)	80 (15.4)	80 (14.8)		3.75–3.75	1
Median GPA (SD)	2 (0.8)	2 (0.8)		0.30–0.30	1
Time to neurosurgical intervention for cerebral metastases in months, median	63	47	1.3	1.01–1.75	**0.001**
Overall survival postoperative in months, median	12	10	1.2	0.82–1.46	**0.020**

Abbreviations: OR: odds ratio, IQR: interquartile range, GPD: graded prognostic Assessment, KPS: Karnofsky performance status scale.

**Fig. 1:**
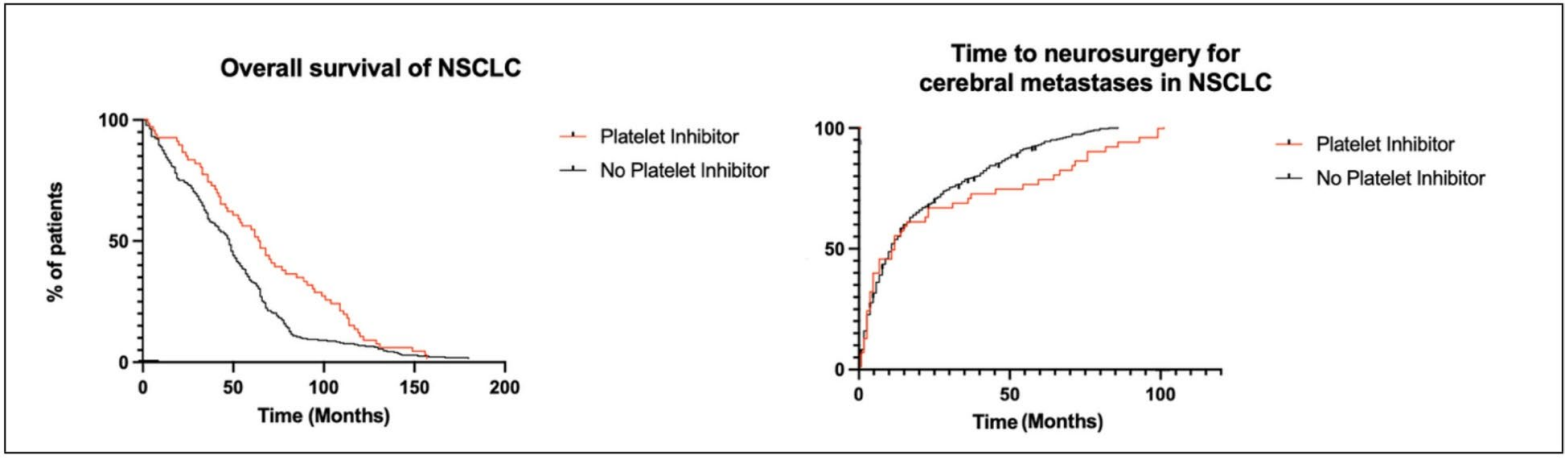
Kaplan-Meier analysis of the effect of platelet inhibitors on time to overall survival (left) and time to neurosurgical intervention for cerebral metastases (right) and in patients with NSCLC and cerebral metastases.

The univariate analysis showed no significant association between patients’ sex or age and platelet inhibitor intake, *p* = 0.195 and 0.513 respectively. The presence of a singular or multiple cerebral metastasis was also non-significant, *p* = 1 and 1.000 respectively. The volumetric analysis of metastasis and its perilesional edema showed no significant association with platelet inhibitor intake (*p* = 0.969 and 0.814). The presence of a hydrocephalus was borderline non-significant according to platelet inhibitor intake (*p* = 0.061). Neither the hemorrhagic transformation nor the hemorrhage were statistically significant *p* = 0.635 and 1.000 respectively. The median KPS was 80 in both the platelet inhibitor cohort and also 80 in patients without platelet inhibitor intake. Furthermore, time to neurosurgical intervention for cerebral metastases showed a strong association with platelet inhibitor intake with 63 months for intake and 47 months for patients without platelet inhibition, *p* = 0.001. In addition, patients with platelet inhibition had an overall survival after surgery (OS-PO) of 12 months in comparison to 10 months (95% CI 0.82–1.46) in patients without platelet inhibition which was also statistically significant, *p* = 0.020. Table 2; Fig. 1. A multivariate analysis confirmed the non-significance of the above analysed parameters.

## Discussion

This study investigated the retrospective effect of the use of platelet inhibitors on the timing of neurosurgical intervention and the risk of intracerebral hemorrhage complications in patients with NSCLC. The most important finding of our study was a significant association between platelet inhibition and delayed need for neurosurgical intervention due to the appearance of brain metastases as well as prolonged survival. At the same time, it is also statistically significant that OS-PO rates are significantly higher in patients receiving antiplatelet therapy.

The clinically significant effect of delayed onset of cerebral metastases in NSCLC is intriguing and the underlying mechanisms are complex. A prerequisite for cerebral metastasis formation is the disruption of the blood-brain-barrier (BBB). The tight junction complex of the BBB is composed of different junctional molecules, including occludin, claudins, and junctional adhesion molecules^8^. Recently, Wei et al., demonstrated their basic research analysis where aspirin was shown to upregulate the expression of tight junction proteins via the TNF-α/HSP70 signaling pathway in a time-dependent manner^9^. This mechanism could in part explain the phenomena observed in our study and aligns with our hypothesis.

In addition, several studies confirmed that coculturing NSCLC cells with human platelets led to the induction of mesenchymal-like cancer cells characterized by downregulation of adhesion proteins, that enhanced cell mobility and a pro aggregatory action on platelets^9^. This effect leads to a coating process that protects circulating tumor cells from the deleterious effects of shear forces^10,11^. Therefore, platelet inhibition hypothetically prevents this effect supporting the observed delayed cerebral metastasis formation in our cohort.

Another mechanism that may explain the improved overall survival in our cohort, could be the direct anti-tumor effectiveness of platelet inhibitors. Several epidemiologic studies have reported an inverse association between aspirin use and the risk of cancer^12^. The anti-neoplastic effect of platelet inhibitors includes the inhibition of COX enzymes that promote carcinogenesis through the synthesis of prostaglandins^13^. Platelet inhibitors have also been shown to upregulate tumor-suppression genes and inhibit NF-kB activation, thus illustrating its anti-cancer activities in a COX independent pathway^14^. On the other hand, mounting preclinical evidence suggests, that platelet inhibitors may exhibit anti-neoplastic effects by inducing apoptosis suppressing angiogenesis, and inhibiting the proliferation of tumor cells^15–17^.

Another clinically relevant finding is the that the use of platelet inhibitors in patients with NSCLC did not cause increased intracranial hemorrhage or hemorrhagic metastasis transformation. This result suggests that the use of platelet inhibitors is safe in patients with NSCLC. This finding is consistent with a recent analysis of Miller et al., where the use of antiplatelet agents was not associated with an increase in the incidence, size, or severity of intracranial hemorrhage in the setting of cerebral metastases^18,19^.

Although not the subject of this study, we must assume that the use of antiplatelets had an underlying cardiovascular indication and that survival was nevertheless better in NSCLC patients with platelet inhibitors. This means that the use of platelet inhibitors in patients with NSCLC can have a positive effect on the prognosis, even if the patients already have a poorer state of health. A prospective study with intake of platelet inhibitors in NSCLC patients without an underlying medical indication could clarify the effect.

### Limitations

While our analysis demonstrates potential benefits of platelet inhibition in a sizable cohort of NSCLC patients, several limitations should be considered. First, the retrospective, multicenter design may introduce variability in clinical management and indications for platelet inhibitor therapy, as well as the risk of confounding, selection bias, and uncontrolled statistical errors. Second, the duration and adherence to platelet inhibitor therapy were not consistently available, which could influence the observed effects. Third, the exact cause of death was not always determined, limiting the interpretation of overall survival outcomes. Finally, the retrospective nature of the study prohibits evaluation of prospective aspects of these effects. Therefore, further prospective randomized trials with large cohorts are needed to validate our findings.

## Conclusions

Our study shows that the use of platelet inhibitors in patients with NSCLC is associated with a delayed need for neurosurgical intervention for cerebral metastases and improved overall survival, without a relevant risk of bleeding.

## Data Availability

The datasets generated during and/or analysed during the current study are available from the corresponding author on reasonable request.
